# Xanthine Oxidase Inhibitor, Allopurinol, Prevented Oxidative Stress, Fibrosis, and Myocardial Damage in Isoproterenol Induced Aged Rats

**DOI:** 10.1155/2015/478039

**Published:** 2015-06-07

**Authors:** Md. Abu Taher Sagor, Nabila Tabassum, Md. Abdullah Potol, Md. Ashraful Alam

**Affiliations:** Department of Pharmaceutical Sciences, North South University, Dhaka 1229, Bangladesh

## Abstract

We evaluated the preventive effect of allopurinol on isoproterenol (ISO) induced myocardial infarction in aged rats. Twelve- to fourteen-month-old male Long Evans rats were divided into three groups: control, ISO, and ISO + allopurinol. At the end of the study, all rats were sacrificed for blood and organ sample collection to evaluate biochemical parameters and oxidative stress markers analyses. Histopathological examinations were also conducted to assess inflammatory cell infiltration and fibrosis in heart and kidneys. Our investigation revealed that the levels of oxidative stress markers were significantly increased while the level of cellular antioxidants, catalase activity, and glutathione concentration in ISO induced rats decreased. Treatment with allopurinol to ISO induced rats prevented the elevated activities of AST, ALT, and ALP enzymes, and the levels of lipid peroxidation products and increased reduced glutathione concentration. ISO induced rats also showed massive inflammatory cells infiltration and fibrosis in heart and kidneys. Furthermore, allopurinol treatment prevented the inflammatory cells infiltration and fibrosis in ISO induced rats. In conclusion, the results of our study suggest that allopurinol treatment is capable of protecting heart of ISO induced myocardial infarction in rats probably by preventing oxidative stress, inflammation, and fibrosis.

## 1. Introduction

Acute myocardial infarction (MI) is one of the most usual diagnosed causes of cardiovascular diseases in hospitalized patients in both developing and developed countries [1, 2]. Myocardial infarction (MI) occurs when there is prolonged imbalance between the myocardial oxygen supply and demand of the myocardium which may in turn results in myocardial necrosis. Inflammatory responses are involved in myocardial tissue damage after an ischemic event. Neutrophil infiltration in the infarct area promotes myocardial cell damage through the release of various cytokines and the production of reactive oxygen species (ROS) [3]. Isoproterenol is a beta-adrenoceptor agonist that induces myocardial infarction by causing imbalance between oxidants and antioxidants in the myocardium [4]. Isoproterenol has been used for a long time to develop experimental animal model for the study of myocardial infarction [5, 6]. Many morphological and biochemical features of the lesions observed following administration of isoproterenol have been characterized. Massive inflammatory cell infiltration and increased cytokines levels were reported in isoproterenol induced model [7, 8]. Among the various other proposed mechanisms for cardiomyocytes damage, the accumulation of free radicals has also been implicated in the pathophysiology of acute myocardial infarction in isoproterenol induced MI animal model [9, 10]. In pathophysiological conditions, the main sources of ROS include the mitochondrial respiratory electron transport chain, xanthine oxidase (XO) activation through ischemia reperfusion, the respiratory burst associated with neutrophil activation, and arachidonic acid metabolism [11].

Xanthine oxidase (XO) is a key enzyme in reactive oxygen species formation. Ischemia-induced cellular calcium overload converts XDH to XO which may produce more ROS, such as superoxide, H_2_O_2_, and hydroxyl radicals [12]. Recent experimental data suggest that XO and XO-related oxidant stress also play a role in the pathogenesis of chronic heart failure [13]. Elevated XO expression and activity have been demonstrated in end-stage human heart failure [14]. Chronic treatment with xanthine oxidase inhibitor, allopurinol, significantly reduced adverse left ventricular remodeling and modestly improved survival of animal included in isoproterenol induced models [15, 16]. However, reversal of myocardial fibrosis and inflammation in isoproterenol induced myocardial infarction by inhibiting xanthine oxidase is not properly investigated yet in aged rats. In view of the protective role of antioxidants against isoproterenol induced myocardial infarction, we have taken up the present research to evaluate the cardioprotective effect of allopurinol against acute myocardial ischemia. We hypothesized that allopurinol has a cardioprotective effect against isoproterenol induced myocardial damage.

## 2. Material and Methods

### 2.1. Animals

Long Evans rats, 12–14 months of age, weighing 250–300 g, obtained from the Animal House of Department of Pharmaceutical Sciences, North South University, Dhaka, Bangladesh, were used in the experiments. The animals were kept at constant temperature (22 ± 2°C), humidity (55%), and light-dark conditions (12/12 h light/dark ratio). The animals were provided with standard laboratory chow diet and drinking water* ad libitum*. The conduct of experiments and the procedure of sacrifice (using Lethabarb) were approved by the Ethics Committee of the Department of Pharmaceutical Sciences, North South University, Dhaka, Bangladesh.

### 2.2. Induction of Myocardial Infarction

Experimental myocardial infarction was induced by injecting isoproterenol (ISO) hydrochloride (dissolved in physiological solution) subcutaneously to rats. To test the effect of allopurinol on isoproterenol induced cardiac disturbances, the animals were divided into three groups. Group one was the control administered saline only and group two was administered isoproterenol (ISO) (at a dose of 50 mg/kg twice a week for two weeks). Group three were treated with both isoproterenol (ISO) (at a dose of 50 mg/kg twice a week for two weeks) and allopurinol (every day at a dose of 100 mg/kg for two weeks). After two weeks, all animals were weighted and sacrificed and we collected the blood and all internal organs such as heart, kidney, spleen, and liver. Immediately after collection of the organs, they are weighted and stored in neutral buffered formalin (pH 7.4) for histological analysis and in refrigerator at −20°C for further studies. Collected blood was centrifuged at 8000 rpm and the plasma separated and stored in refrigerator at −20°C for further analysis.

### 2.3. Assessment of Biochemical Parameters

Liver marker enzymes alanine aminotransferase (ALT), aspartate aminotransferase (AST), and alkaline phosphatase (ALP) were estimated in plasma by using kits obtained from DCI Diagnostics (Budapest, Hungary) according to the manufacturer's protocol. Creatine kinase-MB (CK-MB) activity was also evaluated in plasma using creatine kinase-MB analyzing kit obtained from DCI Diagnostics (Hungary) according to the manufacturer's protocol.

### 2.4. Preparation of Tissue Sample for the Assessment of Oxidative Stress Markers

For determination of oxidative stress markers, heart and kidney tissues were homogenized in 10 volumes of phosphate buffer containing pH 7.4 and centrifuged at 12,000 ×g for 30 min at 4°C. The supernatant was collected and used for the determination of protein and enzymatic studies as described below.

### 2.5. Determination of Lipid Peroxidation (LPO) as Malondialdehyde (MDA)

Lipid peroxidation in tissues was estimated colorimetrically measuring malondialdehyde followed by previously described method [17]. Samples (1 mL) were mixed with 1 mL of 0.67% thiobarbituric acid and placed in a boiling water bath for 10 min. The mixture was cooled and diluted with 1 mL distilled water. The absorbance of the solution was then read using spectrophotometer at 532 nm. The content of malondialdehyde (MDA) (nmol/mL) was then calculated, by reference to a standard curve of MDA solution.

### 2.6. Determination of Nitric Oxide (NO)

Nitric oxide (NO) was determined according to the method described by Tracey et al. as nitrate [18]. In this study, Griess-Ilosvay reagent was modified by using naphthyl ethylenediamine dihydrochloride (0.1% w/v) instead of 1-napthylamine (5%). The reaction mixture (3 mL) containing tissue homogenates or plasma sample (2 mL) and phosphate buffer saline (0.5 mL) was incubated at 25°C for 150 min. A pink colored chromophore was formed in diffused light after addition of modified Griess-Ilosvay reagent. The absorbance of these solutions was measured at 540 nm against the corresponding blank solutions. NO level was measure by using standard curve and expressed as nmol/mL.

### 2.7. Determination of Advanced Protein Oxidation Products (APOP) Assay

Determination of APOP levels was performed by modification of the method of Witko-Sarsat et al. [19] and Tiwari et al. [20]. Two mL of plasma was diluted at 1 : 5 in PBS: 0.1 mL of 1.16 M potassium iodide was then added to each tube, followed by 0.2 mL acetic acid after 2 min. The absorbance of the reaction mixture was immediately read at 340 nm against a blank containing 2 mL of PBS, 0.1 mL of KI, and 0.2 mL of acetic acid. The chloramine-T absorbance at 340 nm was found linear within the range of 0 to 100 nmol/mL; APOP concentrations were expressed as nmol·mL^−1^ chloramine-T equivalents.

### 2.8. Determination of Catalase (CAT) Activity

CAT activities were determined using previously described method by Chance and Maehly [21, 22] with some modifications. The reaction solution of CAT activities contained 2.5 mL of 50 mmol phosphate buffer (pH 5.0), 0.4 mL of 5.9 mmol H_2_O_2_, and 0.1 mL enzyme extract. Changes in absorbance of the reaction solution at 240 nm were determined after one minute. One unit of CAT activity was defined as an absorbance change of 0.01 as units/min.

### 2.9. Determination of Reduced Glutathione (GSH) Assay

Reduced glutathione was estimated by the method of Jollow et al. [23]. 1.0 mL sample of 10% homogenate was precipitated with 1.0 mL of (4%) sulfosalicylic acid. The samples were kept at 4°C for 1 hr and then centrifuged at 1200 ×g for 20 min at 4°C. The total volume of 3.0 mL assay mixture was composed of 0.1 mL filtered aliquot, 2.7 mL phosphate buffer (0.1 M, pH 7.4), and 0.2 mL DTNB (5,5-dithiobis-2-nitrobenzoic acid) (100 mM). The yellow color of the mixture was developed, read immediately at 412 nm on a Smart SpecTM Plus spectrophotometer, and expressed as ng/mg protein.

### 2.10. Histopathological Studies

The heart tissues were fixed in 10% neutral buffered formalin and processed using graded ethanol and xylene treatment. The processed tissues were then embedded in paraffin blocks and sections of about 5 *μ*m thickness were cut by employing a rotary microtome. These sections were stained with Hematoxylin and Eosin using routine procedures. Sirius red staining for fibrosis and Prussian blue staining for iron deposition were also done in heart and kidney sections. Sections were then studied and photographed under light microscope (Zeiss Axio Scope) at 40 magnifications. The slides were examined for pathomorphological changes.

## 3. Results

### 3.1. Effect of Allopurinol Treatment on Body Weight and Organ Wet Weight in ISO Induced Rats

Body weight of rats treated with ISO did not alter significantly compared to control and allopurinol treatment (Table 1). ISO treatment in rats significantly increased the heart and left ventricular wet weight compared to control rats which were ameliorated by allopurinol treatment; however, this change by allopurinol is not statistically significant. Moreover, ISO treatment increased the kidney wet weight and allopurinol did not alter the wet weight of kidney in ISO treated rats.

### 3.2. Effect of Allopurinol Treatment on AST, ALT, and ALP Enzyme Activities in ISO Induced Rats

ISO treatment in rats increased the activities of enzymes such as AST, ALT, and ALP compared to the control rats (Table 2). Allopurinol treatment significantly normalized the elevated AST, ALT, and ALP enzymes activities in ISO treated rats.

### 3.3. Effect of Allopurinol Treatment on CK-MB Activity in ISO Induced Rats

ISO administration in rats increased CK-MB activity in plasma of ISO administered rats compared to control rats (Table 2). Allopurinol treatment significantly decreased the elevated CK-MB enzyme activity in ISO treated rats (Table 2).

### 3.4. Effect of Allopurinol Treatment on Oxidative Stress Parameters in ISO Induced Rats

For studying the oxidative stress parameters, we analyzed MDA, nitric oxide, advanced protein oxidation product (APOP), and GSH level in plasma and tissues. Moreover, we also analyzed catalase enzyme activities in plasma and tissues. ISO treatment in rats showed an increased level of lipid peroxidation product MDA in plasma and tissues compared to the control rats (Table 2). ISO treatment also increased nitric oxide and advanced protein oxidation product in plasma and tissues compared to control rats. Moreover, ISO treatment in rats decreased antioxidant enzyme catalase activities in plasma and tissues compared to control rats. Allopurinol treatment prevented the rise of lipid peroxidation product MDA, NO, and APOP concentration in both plasma and tissues (Table 2). Allopurinol treatment also restored the antioxidant enzyme catalase activities in plasma and tissues near normal compared to control rats.

### 3.5. Effect of Allopurinol Treatment on Histological Assessments in Heart and Kidney Structure in ISO Induced Rats

**Figure 1:**
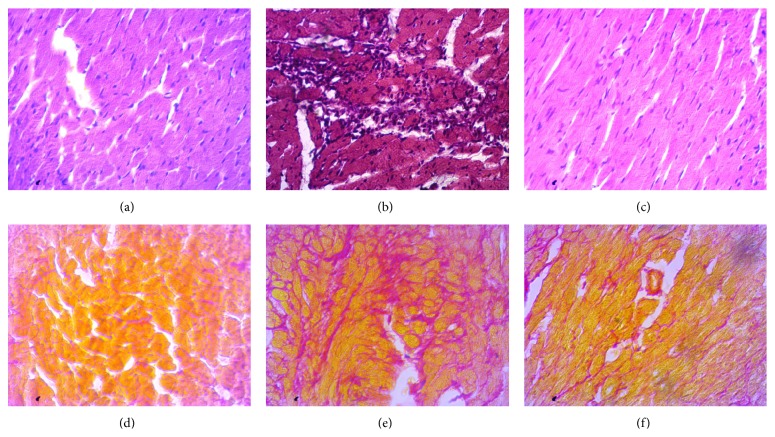
Hematoxylin and Eosin staining showed inflammatory cell infiltration and necrosis in left ventricle of heart section of rats treated with ISO. Control rats showed normal architecture with very well-shaped cardiomyocytes in left ventricle of heart (a). ISO treatment increased necrosis to cardiomyocytes and the mononuclear inflammatory cells infiltration in left ventricle of heart (b) which was normalized by allopurinol treatment (c). Moreover control rats showed normal baseline collagen around cardiomyocytes (a) which was significantly increased in ISO treated rats (b). Allopurinol treatment further prevented the fibrosis in heart of ISO treated rats. Sirius red staining showed fibrosis (red color) in left ventricle of heart section of rats treated with ISO. (a, d) Control; (b, e) ISO; and (c, f) ISO + allopurinol, magnification 40x.

**Figure 2:**
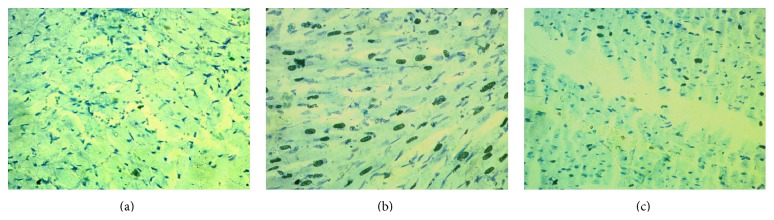
Control rats showed low amount of mast cells in LV of heart (a). However, ISO administration increased degranulated mast cell infiltration in LV of heart which was significantly reduced in allopurinol treated rats. Toluidine blue staining showed mast cells infiltration (deep blue dots) in heart of rats treated with ISO. (a) Control; (b), ISO; (c) ISO + allopurinol, magnification 40x.

**Figure 3:**
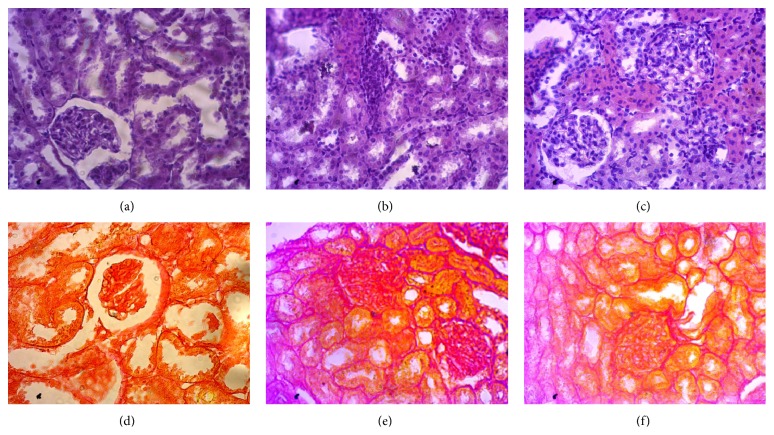
Hematoxylin and Sosin staining showed inflammatory cell infiltration and necrosis in kidney section of rats treated with ISO. Sirius red staining showed fibrosis (red color) in kidney section of rats treated with ISO. (a, d) Control; (b, e) ISO; and (c, f) ISO + allopurinol, magnification 40x.

**Figure 4:**
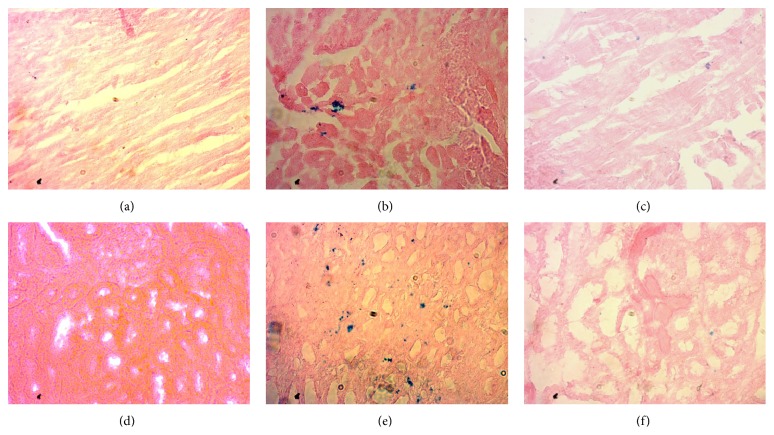
Prussian blue staining showed iron deposition in heart and kidney section of rats treated with ISO and allopurinol. (a, d) Control; (b, e) ISO; and (c, f) ISO + allopurinol, magnification 40x.

Histological staining showed massive mononuclear inflammatory cells infiltration in heart and kidney of ISO induced rats compared to control rats (Figures 1 and 3). Allopurinol prevented the inflammatory cell infiltration in heart and kidney of ISO treated rats. Moreover, mast cells infiltration was also observed in ISO induced rats which were ameliorated in allopurinol treated rats (Figure 2). ISO induced rats also showed hypertrophy of cardiomyocytes and fibrosis along with the inflammation compared to control rats. Cardiomyocytes hypertrophy and fibrosis were also ameliorated by allopurinol treatment (Figure 1). ISO treatment also increased the iron deposition in heart and kidney of rats compared to control rats (Figure 4) which are further ameliorated by allopurinol treatment in ISO induced rats.

## 4. Discussion

Ageing is a degenerative process where important physiological processes are declined and aged individuals generally suffer cardiovascular dysfunction, diabetes, and neurological disorder. However most of the preclinical studies related to cardiovascular outcome are based on young animal studies. In the current study, subcutaneous administration of isoprenaline induced “infarct-like” lesions in the heart of aged rats similar to those present in MI in humans [24]. We also found that the treatment with allopurinol ameliorated the oxidative stresses, inflammation in heart and kidneys of ISO treated aged rats. Rats treated with ISO have been reported to undergo increase in heart wet weight [25]. Hypertrophy of hearts as well as cardiomyocytes was also observed in this rat model. One postulate proposed about increased heart wet weight is due to increase in water content and development of oedema in intramuscular spaces culminating in extensive necrotic changes and invasion of inflammatory cells [25]. In our study, identical set of changes were observed in ISO treated rats compared to control rats. Allopurinol treatment significantly prevented the cardiac changes in ISO treated rats.

Reactive oxygen species like superoxide, hydrogen peroxide, and malondialdehyde (MDA) are produced in enormous amount which contribute to myocardial tissue injury during myocardial infarction [26]. Autooxidation of isoproterenol results in excessive formation of free radicals and lipid peroxidation which causes an irreversible damage to heart [27]. This free radical-mediated peroxidation of membrane phospholipids leads to permeability changes in the myocardial membrane, intracellular calcium overload, and irreversible damage [28]. Moreover, xanthine oxidase participates in a great part of free radical generation in infracted heart [29]. Xanthine oxidase catalyses the conversion of hypoxanthine to xanthine, uric acid, and superoxide [29]. In our study, allopurinol administration decreases the levels of lipid peroxidation in isoproterenol treated rats. Similar finding was also reported previously by other investigators [30, 31]. Nowadays, more specific marker of lipid peroxidation products can be determined in plasma and tissues such as 15-F_2t_-isoprostanes [32]. However, one of the limitations of our study was not to measure 15-F_2t_-isoprostanes due to lack of logistic support. Several studies showed that 15-F_2t_-isoprostanes level increases in presence of free radical mediated oxidative stress [31, 32].

Injury to heart tissues also results in the release of ALT and AST which are present in cardiac muscle and can be found in blood stream [33]. These enzymes are also increased in myocardial infarction. Increased activities of ALT and AST were found due to the leakage of these enzymes as a result of necrosis induced by ISO in rats. Allopurinol treatment in our study showed decreased activities of AST and ALT enzyme in plasma. Our investigation also showed that creatine kinase-MB (CK-MB) activity elevated significantly in plasma of ISO administered rats compared to control rats and confirming the acute myocardial infarction. CK-MB is localized predominantly in the heart and this makes it a valuable diagnostic tool for MI since damage specific to the myocardium would result in elevation of CK-MB levels [34]. These observations are in line with previous studies done on rats treated with isoproterenol [35, 36]. Treatment with allopurinol significantly decreased the CK-MB activity in plasma of ISO administered rats.

Nitric oxide level was also found to be increased in ISO treated rats. It has been reported that inducible nitric oxide synthase (iNOS) expression and nitric oxide (NO) production increase in the myocardial infarcted heart [37]. *β*-Adrenergic stimulation also upregulated iNOS and significantly increases production of NO [38]. Increased nitric oxide concentration creates a nitrosative stress in presence of other reactive oxygen species (ROS) such as superoxides and generates the powerful oxidant molecule peroxynitrite (ONOO-). Inhibition of superoxide production may be beneficial in peroxynitrite production. In our study, allopurinol treatment prevented the rise of nitric oxide level in ISO treated rats.

Antioxidants constitute the defense mechanism that limits the free radicals which initiated damage in tissues. Free radical scavenging enzymes such as SOD, CAT, and GPx are the first line cellular defense enzymes against oxidative stress and nitrosative stress [39]. Increased lipid peroxidation decreases these enzymes in tissue level [40]. ISO induced myocardial damage is also associated with decreased endogenous antioxidants such as superoxide dismutase (SOD) and catalase in heart tissue which are structurally and functionally impaired by free radicals resulting in damage to myocardium [41, 42]. In this study, a significantly lower activity of CAT in heart and kidney tissue and decreased level of reduced glutathione (GSH) in plasma were observed in ISO administered rats when compared to control rats. Further, allopurinol treatment normalized the catalase activity and GSH level in ISO treated rats.

In the present study, the left ventricles of ISO group showed widespread myocardial structure abnormalities and endocardial necrosis related to cardiac tissue edema and myofibrillar fracture. Free radical mediated cellular damage also develops inflammatory response in tissues [43]. ISO treatment also induces massive serge of inflammatory cell infiltration in heart and kidney section compared to control rats. Histopathological examination of myocardial tissue in allopurinol treatment in ISO induced rats illustrated improved integrity of the myocardial cell membrane with decreased focal necrosis and inflammatory cell infiltration when compared to the ISO induced heart. Previous study suggests that monocytes and neutrophil and macrophages infiltration occurred in failing myocardium [44]. Inflammatory cells are generally contributed to extracellular matrix (ECM) deposition in tissues and initiates fibrosis [45, 46]. In our study we also found that mast cells are infiltrated in myocardium of ISO treated rats. Cardiac mast cell population has strong positive correlation with collagen deposition in hypertrophic heart [47]. It has also been reported that cardiac mast cell density increased dramatically with age in the SHR [48]. In this study, allopurinol treatment prevented the extracellular matrix (ECM) deposition in heart and kidneys of ISO treated rats. Similar cardioprotective and antifibrotic activity of allopurinol treatment was also found in other studies where allopurinol prevented pathological remodeling of the heart and fibrosis in diabetic rats [31], AngII induced hypertensive mice [30], and L-NAME induced cardiomyopathy in rats [49].

In conclusion, our study reveals that allopurinol exerts significant cardioprotective effect against ISO induced myocardial infarction in aged rats. This protective effect could be associated with the enhancement of antioxidant defense system and attenuation of inflammatory cells infiltration in the myocardium.

## Figures and Tables

**Table 1:** Effect of allopurinol on body weight and organ weight of isoprenaline treated rats.

Parameters	Control	ISO	ISO + allopurinol
Initial body weight	250.50 ± 12.54	275.35 ± 15.69	261.42 ± 15.24
Final body weight	267.40 ± 13.54	272.60 ± 272.60	274.57 ± 13.65
Kidneys wet weight	0.60 ± 0.02	0.66 ± 0.02	0.63 ± 0.01
Heart wet weight	0.24 ± 0.01	0.35 ± 0.01	0.31 ± 0.01
LV wet weight	0.19 ± 0.01	0.28 ± 0.01	0.25 ± 0.01
RV wet weight	0.09 ± 0.01	0.12 ± 0.01	0.12 ± 0.01

Values are presented as mean ± SEM. *N* = 5–7 in each group or otherwise specified. One-way ANOVA with Bonferroni tests were done as post hoc test. Values are considered significance at *p* < 0.05.

**Table 2:** Effect of allopurinol on biochemical parameter in plasma, heart, and kidney of isoprenaline treated rats.

Parameters	Groups		
	Control	ISO	ISO + allo.
Plasma			
AST (U/L)	27.56 ± 3.22^a^	55.13 ± 2.11^b^	40.20 ± 4.80^a^
ALT (U/L)	29.29 ± 3.45^a^	49.96 ± 6.33^a^	35.89 ± 6.06^a^
ALP (U/L)	58.28 ± 5.92^a^	80.35 ± 5.58^ab^	54.89 ± 6.12^ac^
MDA (nmol/mL)	38.77 ± 1.84^a^	64.41 ± 4.89^b^	41.72 ± 1.68^a^
NO (nmol/mL)	3.87 ± 0.27^a^	6.80 ± 0.65^b^	5.44 ± 0.54^a^
APOP (nmol/mL equivalent to chloramine-T)	228 ± 10.68	656.90 ± 101.18	390.63 ± 61.52
Catalase (U/min/mg protein)	20.83 ± 3.52	14.17 ± 2.01	41.67 ± 6.79
GSH (ng/mg protein)	11.48 ± 0.52^a^	7.55 ± 0.40^b^	13.33 ± 1.39^c^
CK-MB (U/L)	38.89 ± 7.78^a^	120.56 ± 15.26^b^	62.22 ± 7.78^a^
Heart			
NO (nmol/mL)	14.12 ± 0.91^a^	15.87 ± 2.23^a^	14.45 ± 0.65^a^
MDA (nmol/mL)	36.08 ± 0.81^a^	48.51 ± 2.41^b^	45.00 ± 1.00^b^
APOP (nmol/mL equivalent to chloramine-T)	632.06 ± 33.84	706.67 ± 44.95	655.87 ± 24.60
Catalase (U/min/mg protein)	154.17 ± 17.77	68.33 ± 8.33	231.67 ± 25.35
Kidney			
NO (nmol/mL)	22.59 ± 1.66^a^	29.19 ± 3.11^a^	23.51 ± 2.32^a^
MDA (nmol/mL)	39.03 ± 1.04^a^	53.26 ± 2.08^b^	39.00 ± 0.00^a^
APOP (nmol/mL equivalent to chloramine-T)	687.62 ± 23.80^a^	786.83 ± 122.40^a^	727.30 ± 48.75^a^
Catalase (U/min/mg protein)	38.33 ± 4.22	17.50 ± 1.12	40.83 ± 4.73

Values are presented as mean ± SEM. *N* = 5–7 in each group or otherwise specified. One-way ANOVA with Bonferroni tests were done as post hoc test. Values are considered significant at *p* < 0.05. a versus b: control versus ISO; b versus c: ISO versus allopurinol treatment.
